# Artificial double inversion recovery images for (juxta)cortical lesion visualization in multiple sclerosis

**DOI:** 10.1177/13524585211029860

**Published:** 2021-07-14

**Authors:** Piet M Bouman, Victor IJ Strijbis, Laura E Jonkman, Hanneke E Hulst, Jeroen JG Geurts, Martijn D Steenwijk

**Affiliations:** Department of Anatomy & Neurosciences, MS Center Amsterdam, Amsterdam Neuroscience, Amsterdam, The Netherlands/Amsterdam UMC, Vrije Universiteit Amsterdam, Amsterdam, The Netherlands; Department of Anatomy & Neurosciences, MS Center Amsterdam, Amsterdam Neuroscience, Amsterdam, The Netherlands/Amsterdam UMC, Vrije Universiteit Amsterdam, Amsterdam, The Netherlands; Department of Anatomy & Neurosciences, MS Center Amsterdam, Amsterdam Neuroscience, Amsterdam, The Netherlands/Amsterdam UMC, Vrije Universiteit Amsterdam, Amsterdam, The Netherlands; Department of Anatomy & Neurosciences, MS Center Amsterdam, Amsterdam Neuroscience, Amsterdam, The Netherlands/Amsterdam UMC, Vrije Universiteit Amsterdam, Amsterdam, The Netherlands; Department of Anatomy & Neurosciences, MS Center Amsterdam, Amsterdam Neuroscience, Amsterdam, The Netherlands/Amsterdam UMC, Vrije Universiteit Amsterdam, Amsterdam, The Netherlands; Department of Anatomy & Neurosciences, MS Center Amsterdam, Amsterdam Neuroscience, Amsterdam, The Netherlands/Amsterdam UMC, Vrije Universiteit Amsterdam, Amsterdam, The Netherlands

**Keywords:** magnetic resonance imaging, cortical lesions, double inversion recovery, artificial intelligence, clinical trial

## Abstract

**Background:**

Cortical lesions are highly inconspicuous on magnetic resonance imaging (MRI). Double inversion recovery (DIR) has a higher sensitivity than conventional clinical sequences (i.e. T1, T2, FLAIR) but is difficult to acquire, leading to overseen cortical lesions in clinical care and clinical trials.

**Objective:**

To evaluate the usability of artificially generated DIR (aDIR) images for cortical lesion detection compared to conventionally acquired DIR (cDIR).

**Methods:**

The dataset consisted of 3D-T1 and 2D-proton density (PD) T2 images of 73 patients (49RR, 20SP, 4PP) at 1.5 T. Using a 4:1 train:test-ratio, a fully convolutional neural network was trained to predict 3D-aDIR from 3D-T1 and 2D-PD/T2 images. Randomized blind scoring of the test set was used to determine detection reliability, precision and recall.

**Results:**

A total of 626 vs 696 cortical lesions were detected on 15 aDIR vs cDIR images (intraclass correlation coefficient (ICC) = 0.92). Compared to cDIR, precision and recall were 0.84 ± 0.06 and 0.76 ± 0.09, respectively. The frontal and temporal lobes showed the largest differences in discernibility.

**Conclusion:**

Cortical lesions can be detected with good reliability on artificial DIR. The technique has potential to broaden the availability of DIR in clinical care and provides the opportunity of ex post facto implementation of cortical lesions imaging in existing clinical trial data.

## Introduction

Multiple sclerosis (MS) is a chronic inflammatory, demyelinating and neurodegenerative disease of the central nervous system in which cortical grey matter lesions are commonly found.^1–3^ Due to their specificity for MS, cortical lesions were included in the diagnostic criteria.^4^ However, being highly inconspicuous on conventional magnetic resonance imaging (MRI) sequences such as T2 and FLAIR,^5,6^ cortical lesions often remain undetected.

Cortical lesions have been found to be better discernible – with 23% sensitivity – when using double inversion recovery (DIR).^5–8^ In DIR imaging, the signal of the cerebrospinal fluid and white matter are suppressed, such that only signal from grey matter retains. DIR has also been found to be highly pathologically – 91% – specific.^5,7,9^ However, implementation of DIR is often not self-evident in peripheral and even academic hospitals: the sequence is difficult to set up and proper acquisition takes substantial time. Consequently, diagnostic and treatment monitoring processes might be hampered, such as no evidence of disease activity (NEDA) criteria, which are often used as outcome measure for clinical trials.^10–12^

Recent advantages in artificial intelligence enabled medical image-to-image translation, through use of convolutional neural networks and generative adversarial networks.^13–15^ In the current work, we propose to generate artificial DIR (aDIR) images from conventionally acquired 3D-T1 and 2D-proton density (PD)/T2 images. The objective is to evaluate the usability of these aDIR images for cortical lesions detection in patients with MS compared to conventionally acquired DIR (cDIR) images, to broaden availability of DIR in clinical care for diagnosis and treatment monitoring and to create the opportunity for ex post facto implementation of DIR in retrospective image analysis or clinical trials.

## Methods

### Participants

MRI data of 73 patients with MS and 42 healthy controls were retrospectively included for analysis. Radiological, clinical and neuropsychological characteristics of the population were described previously.^16–18^ Patients were all diagnosed with MS according to the McDonald criteria.^19^ Exclusion criteria were contraindications for MR imaging, relapses and/or steroid treatment ⩽6 weeks prior to participation, or presence of neurological and/or psychiatric diseases (other than MS for patients).

### Standard protocol approvals, registrations and patient consents

The studies from which data were included were approved by the institutional ethics committee of Amsterdam UMC, location VUMC, and participants gave written informed consent prior to participation.

### Magnetic resonance imaging

Imaging was performed using a 1.5 T whole-body system (Sonata; Siemens Medical Solutions, Erlangen, Germany) with an eight-channel phased-array head-coil (In Vivo, Orlando, FL). The protocol included a 3D-T1 weighted magnetization-prepared rapid gradient echo (MPRAGE; repetition time (ms) time (ms) 2700/5.03; inversion time (ms) 950; flip angle 8°, sagittal 1.3 mm sections; 1.3 × 1.3 mm^2^ in-plane resolution), a 2D-turbo spin-echo PD/T2 weighed (3130/(24/85), axial 3.0 mm; 1.0 × 1.0) and a 3D-DIR (350/2350, 6500/355; sagittal 1.3 mm; 1.3 × 1.3) sequence.

### Preprocessing

Images were rigidly co-registered with MNI space using FLIRT (part of the FMRIB Software Library (FSL); http://fsl.fmrib.ox.ac.uk). The 3D-T1 weighted sequence of each patient was registered to 1.0 mm isotropic Montreal Neurological Institute (MNI) standard space using FSL’s linear image registration tool (FLIRT) and 12 degrees of freedom (dof). The resulting linear transformation matrix was used to obtain the corresponding rigid registration. Sub-sequently, the rigid transformation matrix was applied to the 3D-T1 weighted image using spline interpolation to transform the data to MNI standard space, and a rough outline of the 3D-T1 brain mask (obtained by ‘betpremask’) was co-registered to MNI standard space. 3D-DIR, PD and T2 weighted sequences were first rigidly aligned to the 3D-T1 sequence in subject space. Resulting rigid transformations were concatenated with the previous transformation matrix to MNI standard space in order to map the other sequences to MNI standard space. All sequences were *Z*-score corrected (mean-shifted and variance-scaled), divided by four normalized between −1 and 1 and interpolated using spline interpolation.

### Network

**Figure 1.**
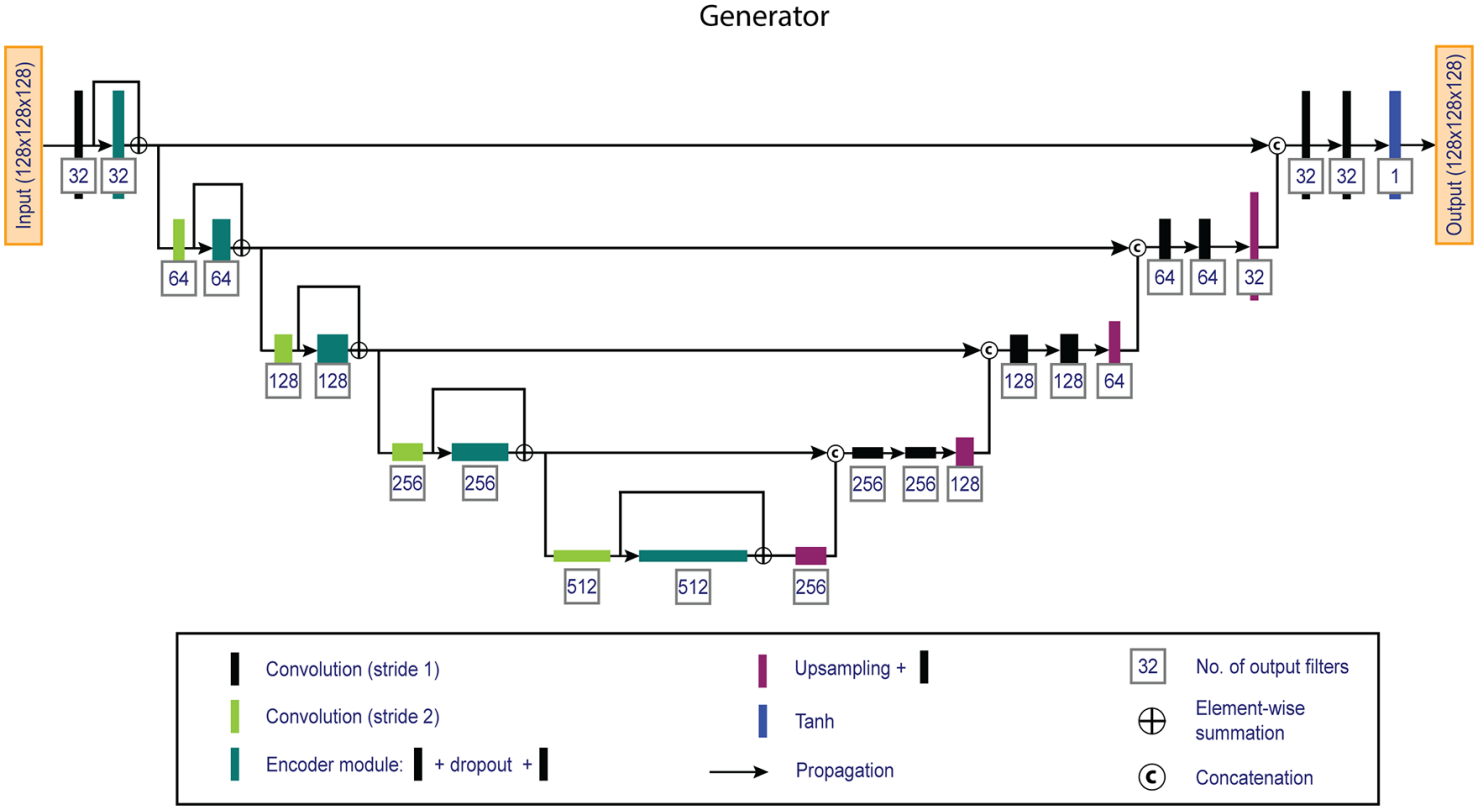
Overview of the 3D fully convolutional generator network. The network consists of an encoder (left) and a decoder (right) pathway that generates artificial double inversion recovery images from standard clinical 3D-T1 and 2D-proton density / T2 weighted images.

**Figure 2.**
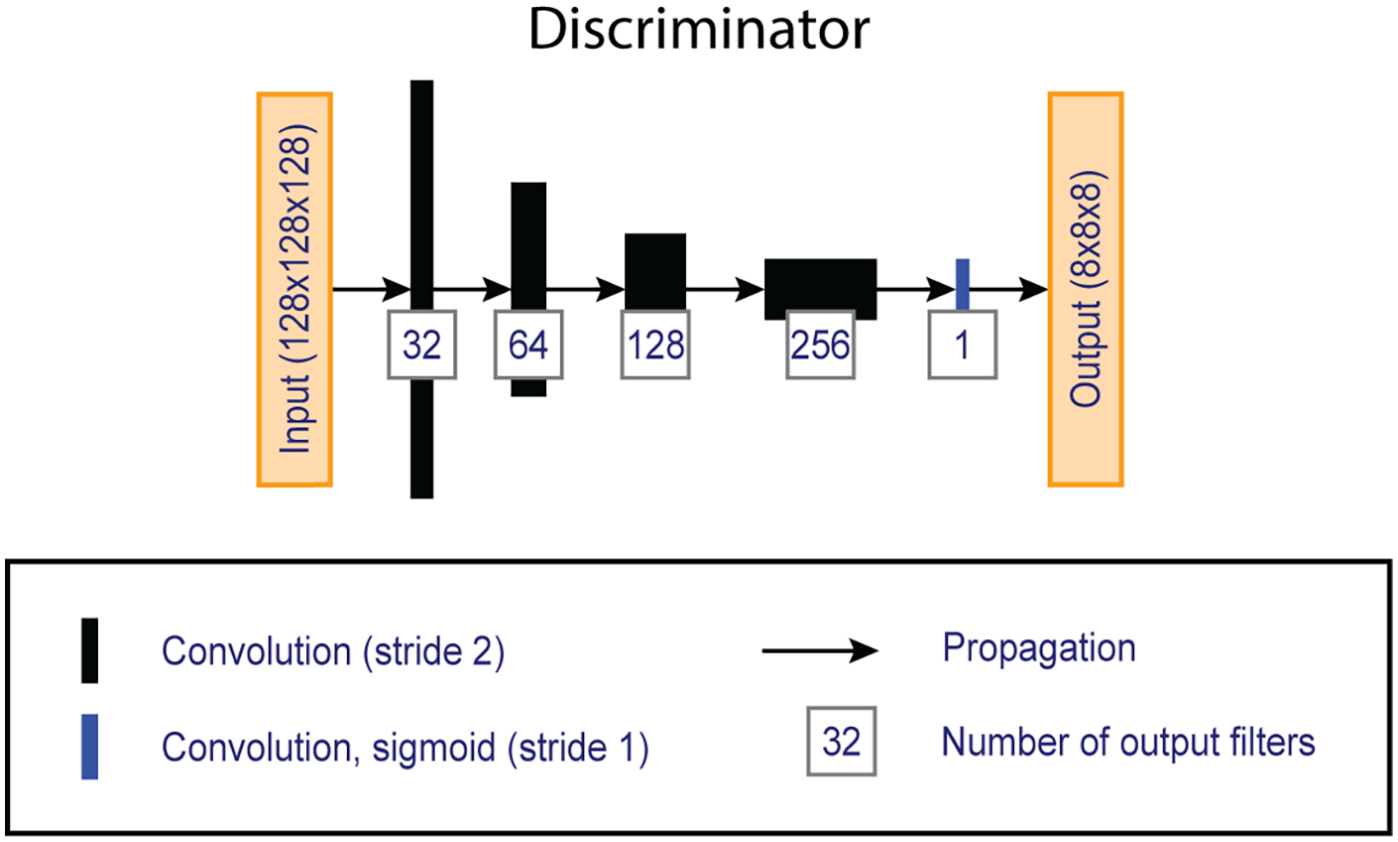
Overview of the ‘PatchGAN’ discriminator network, which compares artificially generated double inversion recovery images with conventionally acquired images given the corresponding clinical images.

The network followed a fully convolutional 3D conditional adversarial design, combining a competing generator and discriminator: the generator (see Figure 1) was trained to produce artificial 3D-DIR images from clinical T1 and PD/T2, while the discriminator (see Figure 2) was trained to discriminate between ‘real’ (cDIR) and ‘fake’ (aDIR) images.^20^

The generator is a U-shaped fully convolutional network that utilizes an encoder and decoder pathway and has been widely established in biomedical imaging tasks.^21^ The generator uses an encoder pathway to encode feature information at decreasing spatial resolutions, and a decoder pathway that combines the encoded feature information with increasing spatial resolutions. The encoder path uses skip-connections to make optimization more efficient.^22^ The network used 128 × 128 × 128 patches, was five layers deep and used 3 × 3 × 3 kernels with 32 filters at the base. Convolutions were followed by instance normalization and Leaky ReLu activations (α = 0.2). A one-strided convolution layer with sigmoid activation provided the validity score.

### Patch selection, data augmentation and model training

Patches – of which the centre voxel was located within the brain mask – were randomly extracted from the train set and augmented to enrich the dataset:

- Mirroring in *x-, y-* and/or *z-*axis, with probability of 0.5 for each axis;
- In 50% of the samples, rotation and scaling in *x-, y-* and/or *z*-axis, with angles and scaling factors randomly sampled from uniform distributions [–90, 90°] and [0.8, 1.2], respectively. Rotation and scaling were combined in a single transformation and applied using three-order spline interpolation (zeroth order for masks).

Predictor only, in 50% of the samples:

- Intensity correction with gamma-value (for each channel) uniformly sampled from the uniform distribution [0.8, 1.5].

Predictor only, in 30% of the samples:

- Additive Gaussian noise (SD = 0.05);
- Gaussian blurring, the standard deviation of the kernel was similar for all channels and randomly sampled from the uniform distribution [0.2, 1.5];
- One image channel randomly zeroed.

Because predictor augmentation altered intensity distribution, they were variance-scaled, divided by 4, normalized between −1 and 1 and clipped just before feeding to the training algorithm.

Model training was performed on a NVIDIA GeForce GTX 1080 TI graphics processing unit using TensorFlow 1.9.0, Cuda 9.0 and python 3.6.9. The number of samples per epoch was determined by floor(sum_of_all_brain_voxels_in_all_train_subjects/128ˆ3)*8. Mean absolute error and binary cross entropy were minimized over 350 epochs with Adam optimizers (initial learning rate = 2e−4, ß_1_ = 0.5, ß_2_ = 0.999). Batch size was 2.

### Model evaluation

Performance was investigated by blinded scoring of cortical lesions (PMB, 3 years of experience and acquainted with MRI appearance of histopathology-validated cortical lesions)^5^ on the artificially generated and conventionally acquired images in the test set (*N* = 23) following consensus recommendations: cortical lesions were identified as areas that were hyperintense compared to normal-appearing grey matter and that were at least 3 mm^2^ in size. Multiple slices were assessed in order to determine whether or not a hyperintensity should be deemed a cortical lesion, as to distinguish between cortical lesions and, for example, cortical vessels, that are round and traceable, or noise.^23^ Furthermore, inter- and intra-rater scores were obtained were calculated between PMB and JJGG (>15 years of experience in scoring cortical lesions). Chance of recognition of the images was minimized by presenting the cases in random order and using left-right mirroring. An additional, retrospective (vis-à-vis) scoring iteration was performed to reduce intra-rater variability and record lobular lesion location and type (cortical, juxtacortical, mixed). Precision (true positives (TP)/(TP + false positives (FP)); fraction lesions on aDIR matching location on cDIR) and recall (TP/(TP + false negatives (FN)); fraction lesions on cDIR detected on aDIR) of cortical lesions detected on aDIR images was calculated taking the cortical lesions detected on cDIR images as reference. Precision and recall measures were calculated for prospective as well as retrospective scoring iterations.

### Statistical analysis

Detection rate (reliability) was measured by calculating intraclass correlation coefficient (ICC; two-way-mixed model in absolute agreement) between the number of detected cortical and infratentorial lesions on aDIR and cDIR images. Inter- and intra-rater scores were also calculated with ICC (two way-mixed model, absolute agreement) between the two scorings. Differences between train and test set were assessed using students’ *t* for normally distributed, Mann–Whitney *U* test for non-normal distributed and chi-square tests for categorical variables. Differences in location and type were analysed using Kruskal–Wallis *H*-tests. Analyses were performed in SPSS 26 (SPSS, Chicago, IL); *p* values <0.05 were considered statistically significant.

## Results

### Demographic characteristics at baseline

Patients’ average age was 46.6 ± 8.1 years (mean ± SD), median [range] EDSS 4.0 [0–7.5], mean disease duration 11.5 ± 6.5 years. Forty-nine patients were diagnosed with relapsing-remitting MS, 20 with secondary progressive MS 4 with primary progressive MS. Mean age of the healthy controls was 45.0 ± 9.1 years.

The dataset was randomly split in train and test set using 4:1 ratio, constituting a train set of 92 (MS/controls: 58/34) cases and a test set of 23 (MS/controls: 15/8) cases. The demographical and clinical characteristics of the train and test set are described to detail in Table 1. Participants in the test set had a shorter disease duration than participants in the train set (*U* = 175.5, *p* = 0.002). Controls in the train set were assessed for presence of cortical lesions, and none were detected.

**Table 1.** Demographic and clinical characteristics^a^.

	Train set		Test set	
	HC	MS	HC	MS
*N*	34	58	8	15
Male sex	12 (35%)	21 (36%)	3 (40%)	3 (20%)
Age (years)	45.0 ± 9.1	49.3 ± 7.6	45.3 ± 9.9	45.6 ± 9.9
Disease duration from diagnosis (years)		12.6 ± 6.1		7.1 ± 6.2*
MS type (RR/SP/PP)		36/18/3		13/2/0
Expanded disability status scale		3.75 (0.0–7.5)		3.5 (2.0–6.0)

HC: healthy controls; MS: multiple sclerosis.

Note. Unless otherwise stated, data are number of participants.

* Indicates statistical significant difference (*p* = <0.05).

a Data are mean ± standard deviation or medians (range).

### Detection rate (reliability), precision and recall

**Figure 3.**
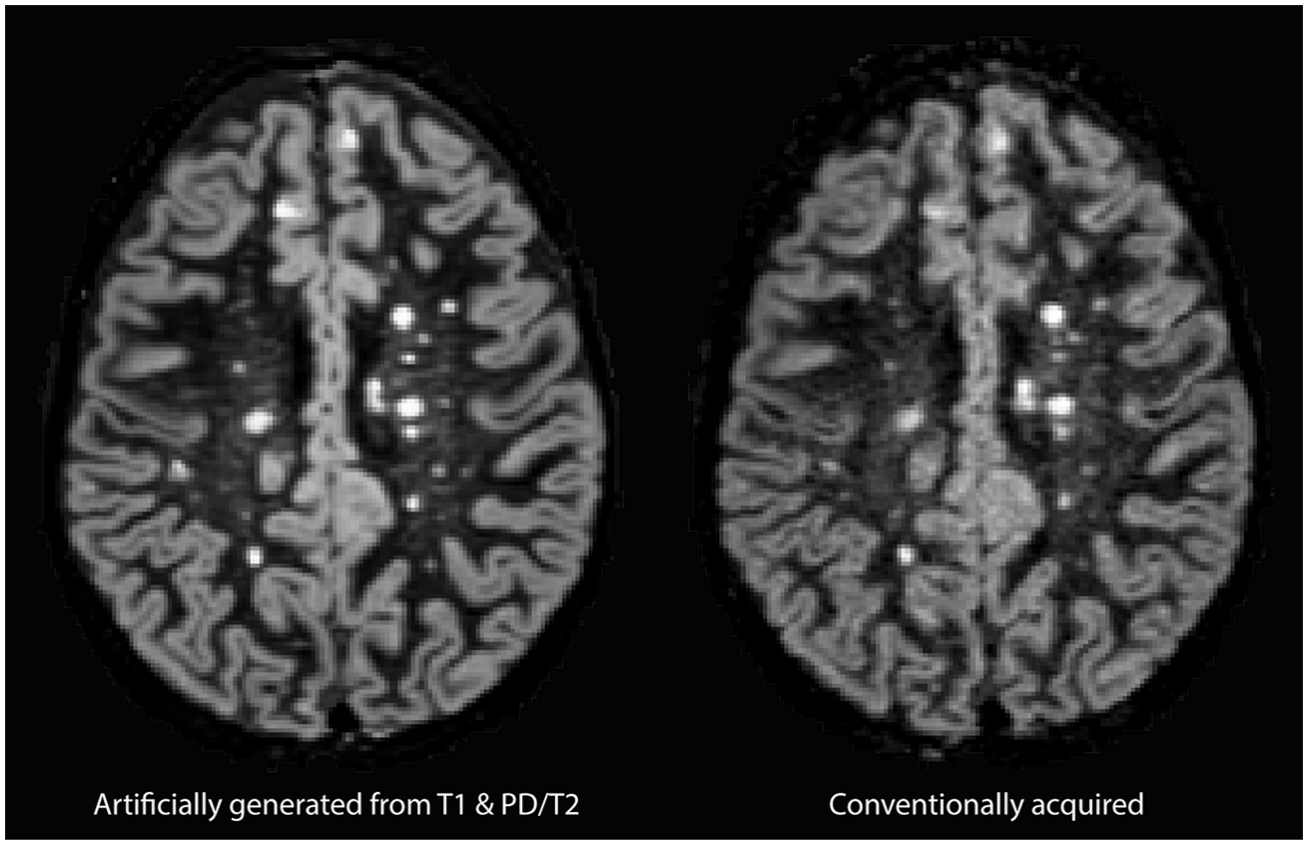
Typical example of artificially generated (left) and conventionally acquired DIR (right) images.

A typical artificially generated and corresponding conventionally acquired DIR image is presented in Figure 3.

**Figure 4.**
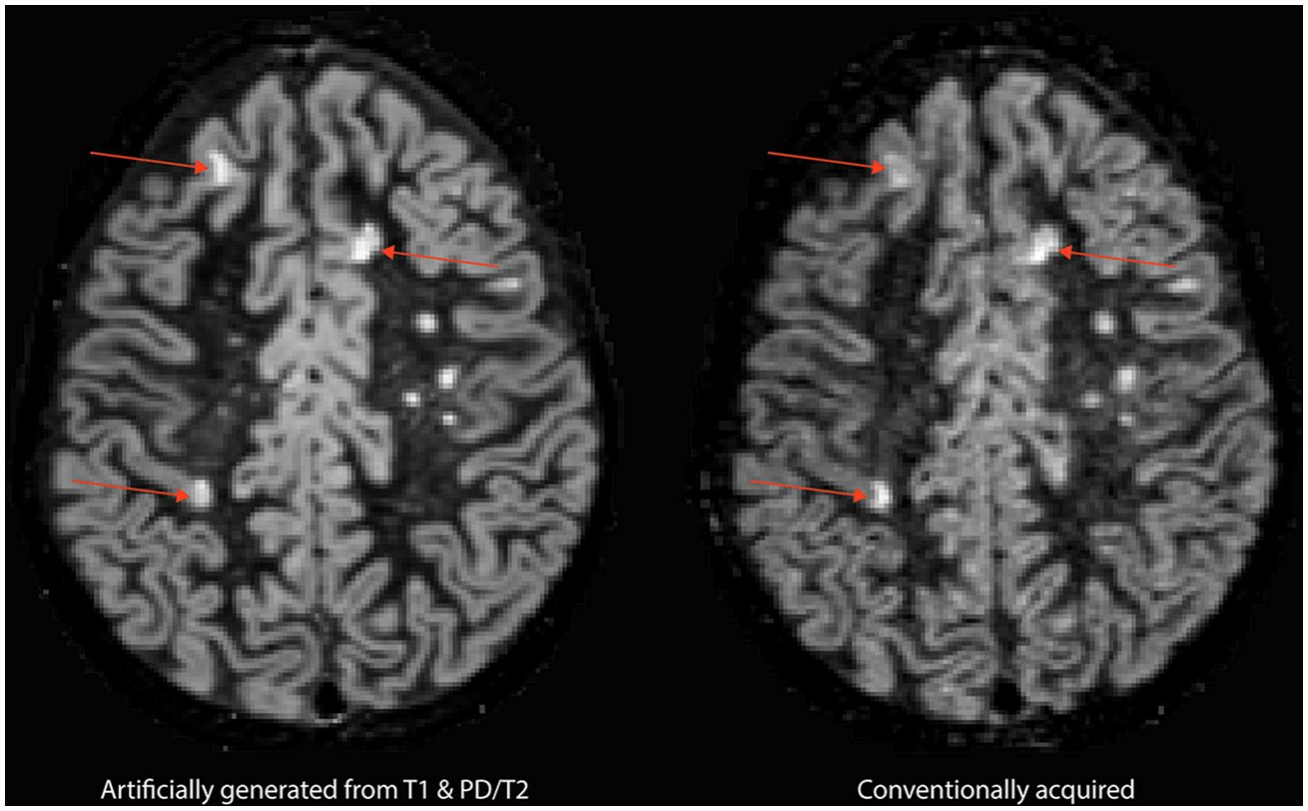
Cortical lesions prospectively visible on both artificially generated DIR images and conventionally acquired DIR.

**Figure 5.**
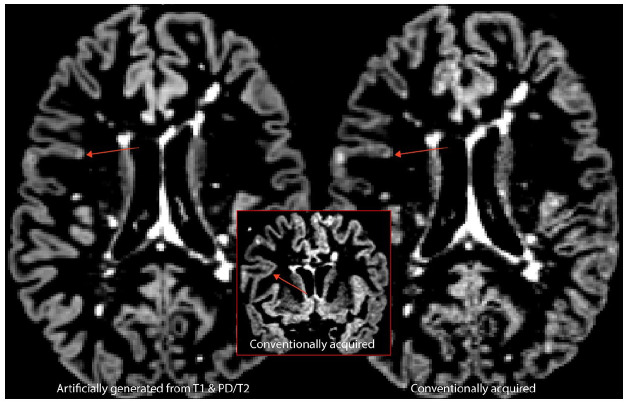
Example of an intracortical lesion prospectively visible in both artificially generated (left) and conventionally acquired (right) DIR images. The inset demonstrates visibility of the intracortical lesion in the coronal plane.

A total of 626 cortical lesions were prospectively detected on aDIR images of the 15 patients in the test set, compared to 696 cortical lesions on the corresponding cDIR images. Of the aDIR-detected cortical lesions, 528 were also detected on cDIR (i.e. ‘true positive’). No cortical lesions were detected in the eight healthy controls in the test set. Reliability analysis showed a high agreement in the number of detected cortical lesions between aDIR and cDIR (ICC = 0.917, 95% CI = 0.675–0.975 (*F*(32.755)), *p* < 0.001). Subsequent analysis of infratentorial lesions showed high agreement as well (ICC = 0.855, 95% CI = 0.589–0.954 (*F*(11.935)), *p* < 0.001). Figure 4 provides an example of an aDIR image and its cDIR counterpart with the detected cortical lesions in it. Figure 5 highlights an intracortical lesion that was detected in the data on both sequences. Intra-rater ICC score for aDIR was 0.991, intra-rater ICC score for cDIR was 0.984. Inter-rater scores were 0.890 for aDIR and 0.862 for cDIR.

The average precision of the detected cortical lesions on aDIR images compared to cDIR images was (mean ± SD) = 0.85 ± 0.06, ranging from 0.72 to 0.91. The average recall of cortical lesions detected on aDIR images compared to cDIR images was 0.76 ± 0.09, ranging from 0.57 to 0.92.

The unblinded retrospective scoring iteration revealed a total of 679 cortical lesions on aDIR images (i.e. 53 more) and 722 cortical lesions on cDIR images (i.e. 26 more). Reliability of the retrospective data showed an even higher agreement in number of detected cortical lesions between aDIR and cDIR (ICC = 0.936, 95% CI = 0.817–0.978 (*F*(33.714)), *p* < 0.001) as well as for the infratentorial lesions (ICC = 0.952, 95% CI = 0.855–0.985 (*F*(38.974)), *p* < 0.001). Retrospective precision and recall were measured at 0.76 ± 0.09, ranging from 0.50 to 0.86, and 0.73 ± 0.10, ranging from 0.49 to 0.89, respectively. Of the 168 cortical lesions that were prospectively visible on cDIR images, but not on aDIR images, 53 were retrospectively discernible (31.5%). Of the 98 cortical lesions that were initially visible on aDIR images but not on cDIR images, 30 were visible upon retrospective assessment (30.6%).

### Lesion distribution and type

An overview of the (lobular) cortical distribution and lesions type (i.e. intracortical, juxtacortical or mixed) of the prospectively detected lesions is provided in Table 2. The vast majority of lesions that were visible on both the aDIR and cDIR images was located in the frontal lobe, followed by temporal, parietal and occipital lobes. The vast majority were intracortical, followed by juxtacortical and mixed lesions. On the contrary, a total of 98 cortical lesions was prospectively detected on aDIR images but not on cDIR images. These cortical lesions were predominantly situated in the frontal lobe, followed by temporal, parietal and occipital lobes.

**Table 2.** Overview of prospectively detected cortical lesions on artificially generated and conventionally acquired DIR images.

Sequence	Conventionally acquired and artificially generated DIR	Artificially generated DIR^a^	Conventionally acquired DIR^b^
**N cortical lesions**	528	98	168
**Lobular location**			
** Frontal**	245 (46.4%)	42 (42.9%)	80 (47.6%)
** Temporal**	181 (34.3%)	34 (34.7%)	60 (35.7%)
** Parietal**	97 (18.4%)	20 (20.4%)	22 (13.1%)
** Occipital**	5 (0.9%)	2 (2.0%)	6 (3.6%)
**Lesion type**			
** Intracortical**	393 (74.4%)	81 (82.7%)	121 (72.0%)
** Juxtacortical**	103 (19.5%)	10 (10.2%)	36 (21.4%)
** Mixed**	32 (6.1%)	7 (7.1%)	11 (6.5%)

DIR: double inversion recovery.

Note. Numbers indicate detected cortical lesions.

a Lesions were not detected on conventionally acquired DIR images.

b Lesions were not detected on artificially generated DIR images.

## Discussion

Cortical lesions play a pivotal role in the pathophysiology of multiple sclerosis, while implementation of the tools for appropriate assessment (i.e. availability of DIR images) is not self-evident in many hospitals, since they are not readily available on all clinical systems and acquisition of the sequence itself is time-consuming. Heretofore, cortical lesion detection was mostly performed using conventional clinical T1, T2 and FLAIR sequences, which have been found to be less sensitive than DIR.^5^ In this work, we evaluated the usability of artificial DIR images, generated from conventional clinical 3D-T1 and PD/T2 sequences, for cortical lesion detection in multiple sclerosis patients set against a conventionally acquired DIR sequence.

The main finding of this study is that cortical lesions can be detected on aDIR images, with high reliability compared to cDIR images. Our results displayed a prospective ICC cortical lesion count of >0.9, which can be considered excellent.^24^ In addition, we found that 76% of cortical lesions that were detected on cDIR are also discernible on aDIR, of which 84% also match location with cDIR. Furthermore, there were no differences in number of detected lesions per lobe and lesion type between aDIR and cDIR, underlining the high similarity between the sequences. DIR has been found to have the tendency of overdiagnosing intracortical lesions when set against heavily T1 weighted sequences such as PSIR.^25^ Diagnostic criteria for MS are, however, not specific on the cortical lesion subtype (i.e. cortical or juxtacortical).^4^ Hence, this equivocality has no consequences for the usability of aDIR images. The relatively high number of detected lesions could be attributed to (1) the raters being acquainted with MRI-appearance of histopathologically validated cortical lesions and (2) lesions in close proximity that were counted apart but actually form part of the same lesion being only visible at two points. Moreover, the vast majority of discernible cortical lesions on aDIR were consistent with the literature regarding lesion type and location.^26^ The option to generate aDIR images that are comparable to cDIR images from conventional MRI sequences is promising, since two-third of current clinical trials having MRI lesions or NEDA criteria as outcome measure does not include DIR (www.clinicaltrials.gov). Artificial DIR images provide the opportunity to enhance the results of these studies by adding a DIR sequence based on the (to be) acquired clinical sequences. In addition, clinical trials that have already been finished and have not had the opportunity for optimal cortical lesion detection could be re-interpreted with aDIR images.

There were several lesions detected on aDIR but not cDIR, as reflected in the precision of 84%. Unclear is, whether these are truly false-positives (e.g. hyperintensities that were marked as lesions due to artefacts in image generation), or, whether aDIR images have the potential to visualize cortical lesions that are not discernible on cDIR images. The increased cortical lesion detection on aDIR might also be a consequence of an increased signal-to-noise ratio (SNR) of aDIR compared to cDIR, since SNR has been found to increase cortical lesion discernibility.^5–7,27^ Histopathological validation of the aDIR images would provide clarity on this issue and should be topic of further studies. Furthermore, such would be the final step towards heralding aDIR in clinical care, assessing whether or not its detection rates could be deemed acceptable. Vice versa, analysis of cortical lesions detected on cDIR but not on aDIR showed that some cortical lesions were systematically incorrectly modelled by the convolutional network algorithm. This concerned juxtacortical lesions in close proximity to the dorsal and/or ventral surface of the brain and may be attributed to the fine morphology and high similarity of intra- and extraparenchymal image intensities in these regions.

This work is subject to some limitations. First, all MRI data were gathered on a single scanner, which may have generated particular detectability rates, limiting generalization of the algorithm. Therefore, future works should consider multi-centre validation to generate cross-scanner robustness. Second, next to its acquisition, DIR is difficult to interpret: the sequence is a known host to artefacts and there are stringent criteria for lesion scoring.^23^ Nonetheless, DIR scorings tend to suffer from high inter-rater variability, in particular over different centres. Therefore, clinicians and researchers often consult other sequences for cortical lesion detection. However, many lesions that are discernible on DIR might not be discernible on the other sequences – even with histopathological feedback at hand.^5^ Finally, all aDIR images in the current work were generated from T1 and PD/T2 sequences, while in many hospitals and clinical trials FLAIR is used instead of T2. However, the algorithm is written in such a way that it is also feasible to generate aDIR images based on T1 and FLAIR sequences.

Our results show that it is possible to generate artificial DIR images ex post facto from conventional clinical sequences, which are readily available on any MRI scanner. These artificially generated DIR images allow for similar cortical lesion detection rates as do conventionally acquired DIR images. Further validation of this technique (i.e. histopathological validation and multi-centre cross-scanner robustness) would allow for a wider implementation of DIR in clinical care for diagnosis and treatment monitoring purposes and would enable ex post facto implementation of DIR in retrospective image analysis or clinical trials.
